# Unlocking the novel genetic diversity and population structure of synthetic Hexaploid wheat

**DOI:** 10.1186/s12864-018-4969-2

**Published:** 2018-08-06

**Authors:** Madhav Bhatta, Alexey Morgounov, Vikas Belamkar, Jesse Poland, P. Stephen Baenziger

**Affiliations:** 1 Department of Agronomy and Horticulture, University of Nebraska-Lincoln, 362D Plant Sciences Hall, Lincoln, NE 68583 USA; 2 International Maize and Wheat Improvement Center (CIMMYT), P.K. 39 Emek, 06511 Ankara, Turkey; 3 Wheat Genetics Resource Center, Department of Plant Pathology, Kansas State University, Manhattan, KS 66506 USA

**Keywords:** *Aegilops tauschii*, D-genome diversity, Genotype-by-sequencing, Single nucleotide polymorphism, *Triticum turgidum*, Bread wheat

## Abstract

**Background:**

Synthetic hexaploid wheat (SHW) is a reconstitution of hexaploid wheat from its progenitors (*Triticum turgidum ssp. durum* L.; AABB x *Aegilops tauschii* Coss.; DD) and has novel sources of genetic diversity for broadening the genetic base of elite bread wheat (BW) germplasm (*T. aestivum* L). Understanding the diversity and population structure of SHWs will facilitate their use in wheat breeding programs. Our objectives were to understand the genetic diversity and population structure of SHWs and compare the genetic diversity of SHWs with elite BW cultivars and demonstrate the potential of SHWs to broaden the genetic base of modern wheat germplasm.

**Results:**

The genotyping-by-sequencing of SHW provided 35,939 high-quality single nucleotide polymorphisms (SNPs) that were distributed across the A (33%), B (36%), and D (31%) genomes. The percentage of SNPs on the D genome was nearly same as the other two genomes, unlike in BW cultivars where the D genome polymorphism is generally much lower than the A and B genomes. This indicates the presence of high variation in the D genome in the SHWs. The D genome gene diversity of SHWs was 88.2% higher than that found in a sample of elite BW cultivars. Population structure analysis revealed that SHWs could be separated into two subgroups, mainly differentiated by geographical location of durum parents and growth habit of the crop (spring and winter type). Further population structure analysis of durum and *Ae.* parents separately identified two subgroups, mainly based on type of parents used. Although *Ae. tauschii* parents were divided into two sub-species: *Ae. tauschii ssp. tauschii* and *ssp. strangulate,* they were not clearly distinguished in the diversity analysis outcome. Population differentiation between SHWs (Spring_SHW and Winter_SHW) samples using analysis of molecular variance indicated 17.43% of genetic variance between populations and the remainder within populations.

**Conclusions:**

SHWs were diverse and had a clearly distinguished population structure identified through GBS-derived SNPs. The results of this study will provide valuable information for wheat genetic improvement through inclusion of novel genetic variation and is a prerequisite for association mapping and genomic selection to unravel economically important marker-trait associations and for cultivar development.

**Electronic supplementary material:**

The online version of this article (10.1186/s12864-018-4969-2) contains supplementary material, which is available to authorized users.

## Background

Hexaploid (bread) wheat (*Triticum aestivum* L.) feeds more than one third of the world’s population and is one of the most important staple crops in the world [1]. Bread wheat (BW) evolved from a natural hybridization of the tetraploid cultivated emmer wheat *T. turgidum* L. ssp. *dicoccon* (Schrank) Thell. (2n = 28; AABB, a progenitor of modern durum wheat) with the wild diploid *Aegilops tauschii* Coss. (2n = 14; DD, goat grass) about 8,000 years ago in the Fertile Crescent [2, 3]. Generation of hexaploid wheat from a few accessions of *Ae. tauschii* followed by limited gene flow from *Ae. tauschii* to hexaploid wheat led to limited D-genome diversity [4]. Intercrosses of existing elite wheat germplasm in each breeding cycle and selection has further narrowed the genetic diversity by the depletion of a few alleles from a more diverse gene pool [4]. Such narrow genetic diversity of elite wheat germplasm is a challenge for sustainable wheat production, which is needed for a rapidly growing world population with the predicted dramatic climate changes and other emerging abiotic and biotic stresses.

One approach for broadening the genetic base of BW is utilizing genes from cultivated tetraploid wheat (*T. turgidum*) and from wild relatives (*Ae. tauschii*) through synthetic hexaploid wheat (SHW) production [5–8]. The SHW, often designated as primary synthetic wheat, is a recreation of wheat by crossing between modern durum wheat and wild goat grass. The SHWs provide a rich source of novel genetic diversity [5–8] and often confer resistance to biotic [9] and abiotic stresses [8, 10, 11]. The D-genome from SHW is reported to have higher nucleotide sequence diversity than the D-genome from BW [12]. The lack of sequence diversity in the D-genome of BW can be noted from the number of SNPs identified in the A or B genome which usually ranges from two [13, 14] to five- [15, 16] times higher than SNPs identified in the D genome. Furthermore*, Ae. tauschii* has many desirable genes/alleles for biotic and abiotic stress resistance for wheat improvement [8]. Hence, wheat genome diversity, especially the D-genome diversity, in BW could be improved by crossing to SHW [7]. Introgression of desirable alleles for biotic/abiotic stress resistance and improved end-use quality from wild relatives into elite wheat germplasm is a major objective in many pre-breeding and germplasm development programs [8, 10].

Genetic diversity analysis using amplified fragment length polymorphism (AFLP) [5, 10] and short sequence repeat (SSR) [5, 6, 10] have been reported in SHW, however, genetic diversity and population structure analysis of SHWs using single nucleotide polymorphisms (SNPs) are largely unknown. Also, the SHWs used in this study have not been used previously for genetic studies [11]. Therefore, the objectives of this study were to (i) investigate genetic diversity in unique sets of diverse SHW accessions (101) using SNPs derived from genotyping-by-sequencing (GBS) platform, (ii) decipher the presence of population structure in SHW collection, and (iii) compare genetic diversity among SHWs and 12 elite wheat cultivars (comprising 10 cultivars from Lincoln, Nebraska and two from Turkey) to determine the prospects of broadening the genetic base of BW using SHW. Understanding the genetic diversity and population structure of SHWs will help in effectively using these novel genetic resources in breeding programs to broaden the genetic base of wheat, identify novel genes/genomic regions associated with multiple stresses and useful traits, and utilizing such regions/genes in marker assisted breeding.

## Methods

### Plant material

Initially, 139 SHWs were analyzed for genetic diversity and population structure (Additional files 1 and 2). However, we found 38 of the entries showed misclassification of durum and Ae. parents (Additional files 1 and 2). Therefore, 38 lines were removed from the analysis and remaining 101 entries were used for the genetic diversity and population structure analysis (Additional file 3). Out of 101 SHWs, 15 of them (spring type) originated from one spring durum (Langdon) parent crossed with 15 different *Ae. tauschii* accessions from China, Iran, Kyrgyzstan, Jammu and Kashmir, and Turkmenistan developed by Kyoto University, Japan. The remaining (86) SHW (winter type) originated from the six winter durum parents from Ukraine and Romania (AISBERG, LEUC 84693, PANDUR, UKR-OD 1530.94, UKR-OD 761.93, and UKR-OD 952.92) crossed with 10 different *Ae. tauschii* accessions from Azerbaijan, Iran, Russia, and Unknown (Additional file 3); and they were developed by International Maize and Wheat Improvement Center (CIMMYT) from 2004 to 2013 [11]. Originally, 12 crosses among six durums and 11 *Ae. tauschii* accessions were involved in the creation of 12-winter type SHWs (Additional files 3 and 4). In the early generation of these crosses, due to the segregation, partial sterility and outcrossing, and continuous selection [11], 79 entries were selected as unique lines as they differed phenotypically [11] and on their kinship relationship values (Additional files 5 and 6). Furthermore, we found seven entries (F8 generation) still segregating (possibly due to outcrossing) for spike color and awn characters in the field experiment conducted in 2016 in Konya, Turkey (Additional files 3 and 4), which were selected as new lines and finally resulted in 86 winter SHWs. The SHWs under study have not been well characterized for genetic studies [11] as might be expected with the continued segregation in the lines. The previously known information of these SHWs were provided in Morgounov et al. [11], who documented that the lines had useful genetic variation for multiple diseases resistance including rust resistance (leaf [incited by *Puccinia triticina*], stripe [incited by *P. striiformis*], and stem rust [incited by *P. graminis*]), common bunt (*Tilletia tritici* and *T. laevis*) resistance, barley yellow dwarf virus resistance and resistance to soil-borne pathogens (cereal cyst nematode [incited by *Heterodera avenae*] and crown rot [incited by *Fusarium pseudograminearum*]), and pest resistance including Hessian fly (*Mayetiola destructor*), sunny pest (*Eurygaster integriceps*), and Russian wheat aphid (Diuraphi noxia) resistance. Therefore, these SHWs under study are highly valuable lines for breeding purpose. These SHWs are maintained by the International Winter Wheat Improvement Program (IWWIP) at CIMMYT, Turkey [11]. For genetic diversity comparisons between SHWs and wheat cultivars, 10 elite BW cultivars (‘Camelot’, ‘Cheyenne’, ‘Freeman’, ‘Goodstreak’, ‘Harry’, ‘Overland’, ‘Panhandle’, ‘Robidoux’, ‘Ruth’, and ‘Wesley’) from Lincoln, Nebraska, USA and two BW cultivars (‘Gerek’ and ‘Karahan’) from Turkey were used (Additional file 7).

### Genotyping and SNP discovery

Genomic DNA was extracted from fresh young leaves (approx. 14 days after sowing) using BioSprint® 96 Plant Kit (QIAGEN). The GBS libraries were constructed in 96-plex following digestion with the restriction enzymes PstI and MspI [17] at Wheat Genetics Resource Center at Kansas State University (Manhattan, KS). SNP calling was performed using TASSEL v. 5.2.40 GBS v2 Pipeline [18] with physical alignment to wheat reference genome sequence made available by the International Wheat Genome Sequencing Consortium (IWGSC, RefSeq V1.0) in 2017. The SNPs with MAF less than 5% and missing data more than 20% were removed from the analysis. All lines had more than 80% SNPs called and none were excluded from the analysis. Similarly, for comparing the genetic diversity between SHWs and BW cultivars and analyses specific to the AB or D genomes, GBS derived SNPs were filtered with the same criteria as SHW for genetic diversity analysis.

### Genetic diversity and population structure analysis

Basic genetic diversity summary statistics including: effective number of alleles, observed heterozygosity, heterozygosity within population (gene diversity), standardized measure of population differentiation (F’_ST_) using AMOVA [19], Nei’s standard genetic distance [20], and Jost’s index of population differentiation (Jost’s D) [21], were calculated for SHWs using GenoDive v 2.0b27 program [22]. The details of genetic diversity parameters are provided in GenodDive [22]. Average pairwise divergence or observed nucleotide diversity (π), expected nucleotide diversity or estimated mutation rate (θ) [23], and Tajima’s D [24] were calculated in TASSEL v. 5.2.40 [25]. Evolutionary relationship among SHWs were determined by neighbor joining hierarchical cluster analysis based on genetic similarity in TASSEL [25] and a dendogram was constructed in FigTree V1.4.3 [26]. Analysis of molecular variance was calculated for estimating components of genetic variance among and within population using Arlequin v. 3.5.2.2 [27].

Population structure was inferred using Bayesian clustering algorithm in the program STRUCTURE v 2.3.4 [28] from the command line python program StrAuto [29] and principal coordinate analysis (PCoA) calculated using distance matrix from TASSEL [25]. For identifying the optimal numbers of subpopulations in STRUCTURE and fixation index (F_ST_) of subpopulation, the genotypes were treated as an admixture population with the allele frequencies correlated model with a total of 100,000 burn-in periods followed by 100,000 Markov chain-Monte Carlo iterations for (hypothetical subpopulations) K = 1 to 10 with five independent runs for each K. The structure output was visualized using StructureHarvester [30] and the number of subpopulations were determined from delta K model [31]. Kinship relationship matrix was calculated from centered identity by descent method [32] implemented in TASSEL v. 5.2.40 [25].

## Results

To put these results in perspective, there were seven durum wheat parents and 25 different *Ae.* parents for a total of 101 SWHs. Once the cross is made and the chromosomes are doubled, it would be expected that the SWH should be homozygous. However, our phenotypic data and marker data suggested that heterozygous parents, outcrossing, mechanical mixtures, or misclassification occurred (Additional file 1). This prompted exclusion of 38 lines and the remaining 101 lines were used subsequently in this study (Additional file 2).

**Fig. 1:**
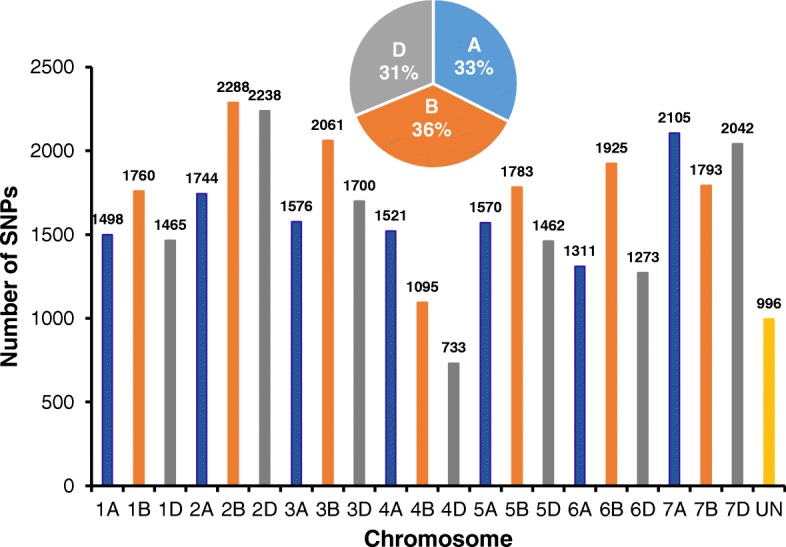
Distribution of 35,939 single nucleotide polymorphisms (SNPs) across 21 chromosomes and unanchored scaffolds in 101 synthetic hexaploid wheats

The GBS derived SNPs were well distributed across the 21 chromosomes in 101 SHWs (Fig. 1). The total number of putative SNPs called from 101 SHWs were 129,115. After filtering, 35,939 SNP markers were used for genetic diversity and population structure analysis (Additional file 8). The B genome had the highest number of SNPs (12,705, ~ 36%), followed by the A genome (11,325, ~ 33%), and the D genome (10,913, ~ 31%). There were 996 SNPs located in scaffolds that are not anchored to any of the chromosomes. The number of SNPs per chromosome ranged from 733 (4D) to 2288 (2B) with an average of 1664 (Fig. 1). The ratio of number of B to A genome SNPs was 1.12, the B to D genome was 1.16, and the A to D genome was 1.04. These ratios indicate the number of SNPs on the D genome were nearly equal to SNPs on the A genome and only slightly lower than SNPs on the B genome.

Summary statistics of various genetic diversity estimates for each genome of SHWs had similar values (Table 1). The average effective number of alleles per locus was 1.54. Observed nucleotide diversity or average pairwise divergence (π bp^− 1^) and gene diversity (Hs) of the SHW genomes were similar and ranged from 0.31 (D genome) to 0.34 (B genome) with an average of 0.33. Expected nucleotide diversity or expected number of polymorphic sites (θ bp^− 1^) and observed heterozygosity (H_o_) in SHWs were similar with an average observed heterozygosity of 0.19. Tajima’s D ranged from 2.04 (D genome) to 2.40 (B genome) with an average of 2.26. Tajima’s D [24] test for selection showed D = 2.26, that means these genotypes showed significant deviation from the neutral expectation (D = 0) and rare alleles were present at low frequencies.

**Table 1:** Distribution of SNP markers and genetic diversity summary statistics of 101 synthetic hexaploid wheats including observed nucleotide diversity (π bp^− 1^), expected nucleotide diversity (θ bp^− 1^), Tajima’s D, effective number of alleles (Eff-num), observed heterozygosity (H_O_), and gene diversity (H_S_)

Genome	No. of SNPs	π bp^− 1^	θ bp^− 1^	Tajima’s D	Eff_Num	H_o_	H_S_
A	11,325	0.33	0.20	2.34	1.55	0.19	0.33
B	12,705	0.34	0.20	2.40	1.56	0.18	0.34
D	10,913	0.32	0.20	2.04	1.51	0.19	0.31
AB	24,030	0.33	0.20	2.38	1.55	0.19	0.33
ABD + Unmapped	35,939	0.33	0.20	2.27	1.54	0.19	0.33

### Population structure

The population structure of 101 SHW was first analyzed on the basis of the ABD genome to study them using all of their genetic diversity. Then the 101 SHWs were analyzed on the basis of the AB and the D genome separately to study genetic diversity of durum and *Ae.* parents, respectively.

#### Population structure of the ABD genome (synthetic hexaploid wheat)

**Fig. 2:**
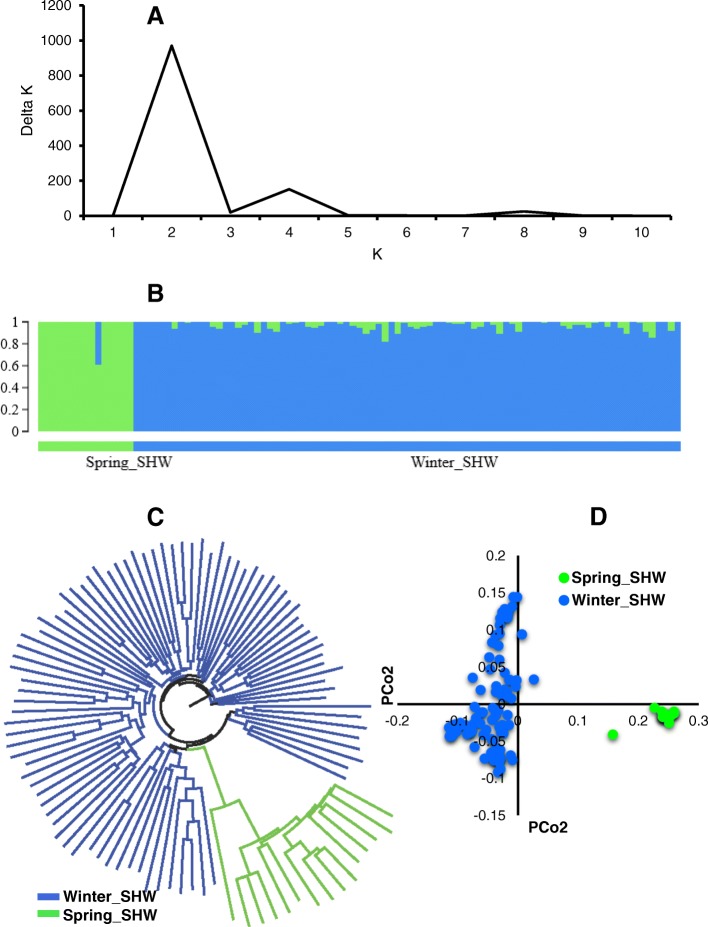
Population strucutre of the 101 synthetic hexaploid wheat germplasm. **a** Line graph of delta K over K from 1 to 10, and the highest peak was observed at Delta K = 2, suggesting the synthetic hexaploid wheat (SHW) germplasm has two subgroups. **b** The two subgroups identified from the STRUCTURE and grouped based on the geographical location of the durum parents and growth habit of the crop. **c** Cluster analysis (neighbor joining) and (**d**) Principal coordinate analysis (PCoA). Color reflects grouping derived from STRUCTURE

The 101 SHWs showed clear evidence of population structure. Delta K values obtained from the STRUCTURE (Bayesian clustering algorithm) output were used to classify subpopulation. The largest delta K was observed at K = 2 (Fig. 2a), suggesting the presence of two subpopulations (Fig. 2b). The first group contains 15 spring SHWs (syn. SHW developed from Japan), designated as ‘Spring_SHW’ and second group contains 86 winter SHWs (syn. SHWs developed by CIMMYT), designated as ‘Winter_SHW’ (Additional file 3). In Spring_SHW, all SHWs (15) had the same durum parent ‘Langdon’ developed in North Dakota, USA. In Winter_SHW, 23 out of 86 SHWs have a durum parent PANDUR developed at Fundulea, Romania and remaining 63 SHWs had durum parents (AISBERG, LECUC.84693, UKR-OD.761.93, UKR-OD.952.92, and UKR-OD.1530.94) developed from Odessa, Ukraine. The growth habit of lines in Spring_SHW were spring type, whereas lines Winter_SHW were winter types.

When comparing the grouping obtained from Bayesian clustering in the neighbor joining cluster (Fig. 2c) and principal coordinate analysis (PCoA) (Fig. 2d), SHWs were again divided into two subgroups (Spring_SHW and Winter_SHW) similar to that of Bayesian clustering (Fig. 2b).

The population structure of SHWs were mainly grouped based on the geographical location of durum parents and growth habit of the crop. Therefore, the population structure using durum and *Ae. tauschii* were studied separately to further understand how durum or *Aegilops* parents were grouped.

#### Population structure using the AB genome (durum parents)

**Fig. 3:**
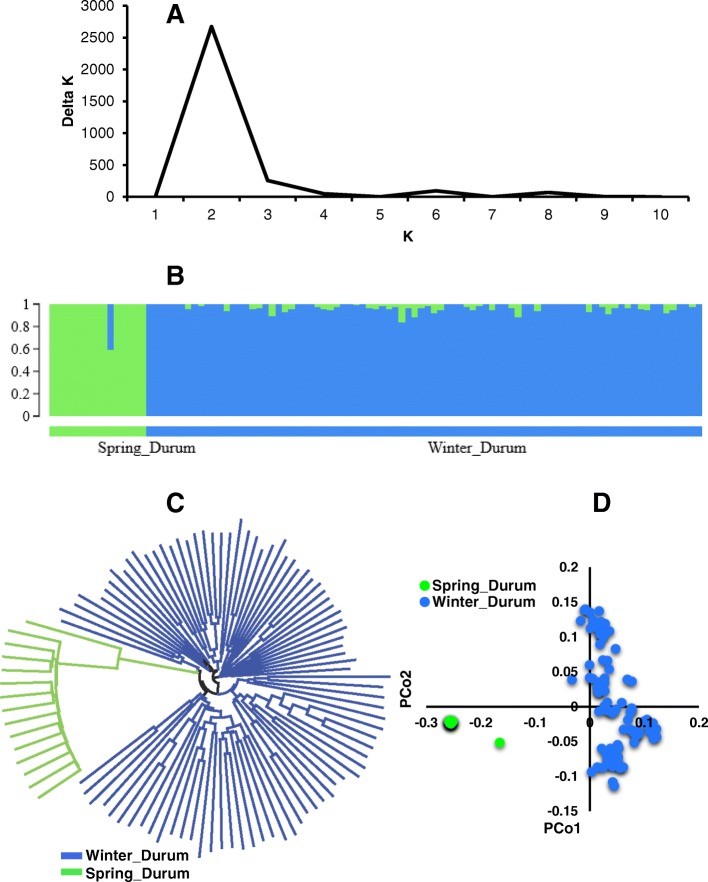
Popualtion structure of the durum parents used in the production of synthetic hexaploid wheat (SHW) germplasm. **a** Line graph of delta K over K from 1 to 10, and the highest peak was observed at Delta K = 2, suggesting the durum wheat used in this study has two subgroups. **b** The two subgroups were identified from the STRUCTURE and grouped based on the geographical location of the durum parents and growth habit of the crop. **c** Cluster analysis (neighbor joining) and (**d**) Principal coordinate analysis (PCoA). Color reflects grouping derived from STRUCTURE

When looking at grouping based on the AB genome (durum parent) of SHWs, two groups were obtained from Bayesian clustering (Fig. 3a and b). The first group contains 15 entries designated as ‘Spring_Durum’ and second group contains 86 entries, designated as ‘Winter_Durum’ (Additional file 3). In Spring_Durum, all entries (15) have a Langdon durum from North Dakota, USA as a parent. In Winter_Durum, 23 out of 86 entries have a durum parent from Romania called PANDUR and remaining 63 entries have a parent from Odessa, Ukraine (AISBERG, LECUC.84693, UKR-OD.761.93, UKR-OD.952.92, and UKR-OD.1530.94). Two subgroups were also classified from the neighbor joining cluster analysis (Fig. 3c) and PCoA (Fig. 3d), and matched the results obtained from Bayesian clustering algorithm (Fig. 2b).

#### Population structure using the D genome (Aegilops parents)

**Fig. 4:**
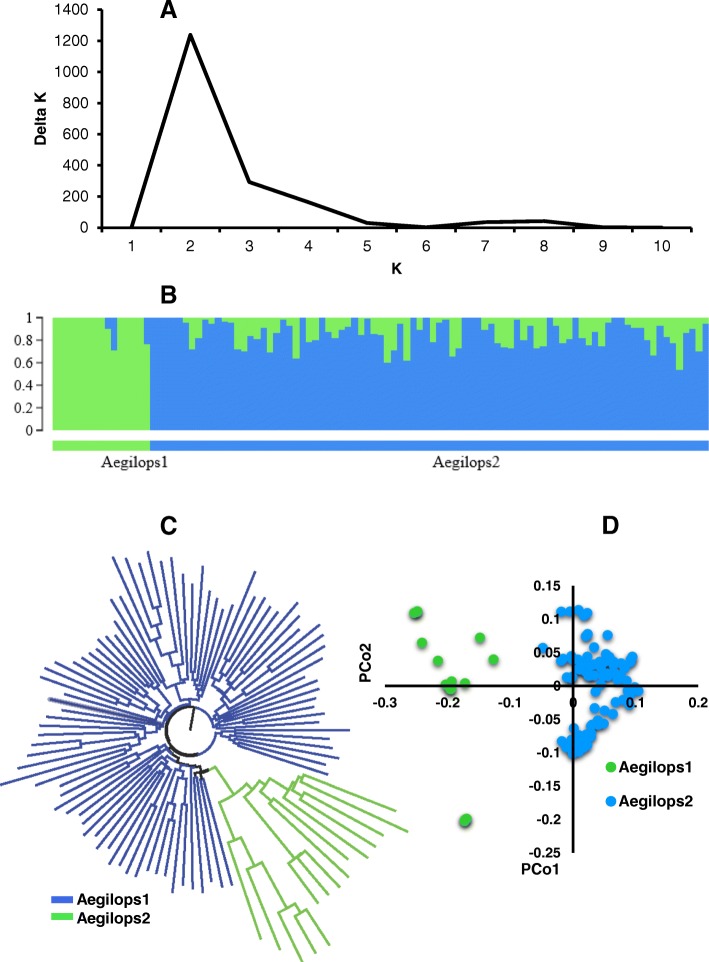
Population strucutre of the *Aegilops* parents used in the production of synthetic hexaploid wheat. **a** Line graph of delta K over K from 1 to 10, and the highest peak was observed at Delta K = 2, suggesting the *Aegilops* used in this study has two subgroups. **b** The two subgroups were identified from the STRUCTURE and grouped based on the type of *Aegilops* parents used. **c** Cluster analysis (neighbor joining) and (**d**) Principal coordinate analysis (PCoA). Color reflects grouping derived from STRUCTURE

When looking at grouping based on the D genome (diploid parent, *Ae. tauschii*) of SHWs, two groups were obtained from Bayesian clustering (Fig. 4a and b). The first group contains 15 entries designated as ‘Aegilops1’ and second group contains 86 entries, designated as ‘Aegilops2’ (Additional file 3). In Aegilops1, 8 out of 15 entries were *Ae. tauschii* ssp. *strangulata* and remaining were *Ae. tauschii* ssp. *tauschii* (2) and unknown (5) (Additional file 3). In Aegilops2, 65 out of 86 entries were *Ae. tauschii* ssp. *tauschii* and remaining were *Ae. tauschii* ssp. *strangulata* (9) and unknown (12) (Additional file 3). Two subgroups were also classified from the neighbor joining cluster analysis (Fig. 4c) and PCoA (Fig. 4d), and matched the results obtained from Bayesian clustering algorithm (Fig. 2b).

### Genetic diversity between the two synthetic Hexaploid wheat groups

The effective number of alleles across SHW groups ranged from 1.31 to 1.51 (Table 2). Observed heterozygosity (H_o_) for the two subgroups identified in Bayesian clustering were similar whereas gene diversity (H_S_) was lower for Spring_SHW (0.18) group compared to Winter_SHW (0.31) group. The F_ST_ (F statistic) obtained from Bayesian clustering measures the divergence and heterogeneity within predefined subgroups and is obtained by estimating the correlation of alleles within the same subgroup [33]. Mean F_ST_ values were about 66% in Spring_SHW and 13% in Winter_SHW, which indicates population differentiation among genotypes in Winter_SHW was lower than Spring_SHW (Table 2). The population differentiation being lower in Winter_SHW indicates the lines are more similar in this subgroup as compared to lines within Spring_SHW.

**Table 2:** Population genetic diversity summary statistics of two subgroups in 101 synthetic hexaploid wheats (SHWs), Durum wheat, and *Aegilops tauschii* (Aegilops) obtained from GenoDive including effective number of alleles (Eff-num), observed heterozygosity (H_O_), and gene diversity (H_S_), and Mean F_ST_ obtained from STRUCTURE

Group	Subgroup	Number of genotypes	Eff_num	H_O_	H_S_	Mean F_ST_
Synthetic Hexaploid Wheat (ABD)						
	Spring_SHW	15	1.31	0.18	0.18	0.66
	Winter_SHW	86	1.51	0.19	0.31	0.13
Durum wheat (AB)						
	Spring_Durum	15	1.22	0.17	0.13	0.79
	Winter_Durum	86	1.54	0.19	0.32	0.10
*Aegilops tauschii* (D)						
	Aegilops1	15	1.49	0.20	0.30	0.30
	Aegilops2	86	1.47	0.19	0.30	0.27

The effective number of alleles across durum subgroups ranged from 1.22 to 1.54 and *Ae.* subgroups ranged from 1.47 to 1.49 (Table 2). Observed heterozygosity within two subgroups of durum and within two subgroups of *Ae.* were similar. Gene diversity of durum subgroups ranged from 0.13 (Spring_Durum) to 0.32 (Winter_Durum) and *Ae.* subgroups (Aegilops1 and Aegilops2) was 0.30.

Pairwise population differentiation was obtained from [34] standardized measure of population differentiation (F’_ST_) estimated using an analysis of molecular variance (AMOVA) [19] and this is used for comparison between organisms with different effective population sizes [34], Jost’s D [21] as an index of population differentiation that is independent of the amount of within population diversity (Hs) computed, and Nei’s D [20] as the standard genetic distance was computed from GenoDive (Table 3). Standardized population differentiation (F’_ST_) between Spring_SHW and Winter_SHW was 0.34, Spring_Durum and Winter_Durum was 0.39, and Aegilops1 and Aegilops2 was 0.22 (Table 3). Similarly, Jost’s D (index of population differentiation) between Spring_SHW and Winter_SHW was 0.17, Spring_Durum and Witner_Durum was 0.20, and Aegilops1 and Aegilops2 was 0.11 (Table 3). The Nei’s D (standard genetic distance) between Spring_SHW and Winter_SHW was 0.19, Spring_Durum and Winter_Durum was 0.22, and Aegilops1 and Aegilops2 was 0.12 (Table 3).

**Table 3:** A standardized measure of population differentiation (F’_ST_), Jost’s D as an index of population differentiation, and Nei’s D as the standard genetic distance in two subgroups in 101 synthetic hexaploid wheats (SHWs) was computed in GenoDive

Population	F’_ST_	Jost’s D	Nei’s D
Spring_SHW and Winter_SHW	0.34	0.17	0.19
Spring_Durum and Winter_Durum	0.39	0.20	0.22
Aegilops1 and Aegilops2	0.22	0.11	0.12

Population differentiation between SHWs (Spring_SHW and Winter_SHW) subgroups using analysis of molecular variance (AMOVA) found that 17.43% of the total genetic variance was explained by the differences between subgroups and 82.57% due to the variation within subgroups (Table 4).

**Table 4:** Analysis of molecular variance (AMOVA) within and between the two subgroups of 101 synthetic hexaploid wheats identified by the Bayesian clustering

Source	d.f.	Sum of squares	Mean squares	Estimated variation	Percentage of variation (%)
Between Populations	1	60,448.12	60,448.12	560.03	17.43
Within populations	99	319,345.46	3225.71	2652.08	82.57
Total	100	379,793.58	–	3212.11	

### Genetic diversity of synthetic Hexaploid wheat and bread wheat cultivars

For comparing the genetic diversity between SHW and BW, 34,887 high quality SNPs available after quality filtering were used for genetic diversity analysis (Additional file 8). The effective number of alleles (Eff-num) was slightly lower for BW (1.26 to 1.38) compared to SHW (1.51 to 1.55) for all genomes (Table 5). The observed heterozygosity of BW (H_O_ = 0.17 to 0.18) was slightly lower compared to SHW (H_O_ = 0.19 to 0.20) for all genomes. The gene diversity was significantly lower for the BW cultivars (H_S_ = 0.17 to 0.25) compared to SHWs (H_S_ = 0.32 to 0.33) for all genomes. Percentage of SHW gene diversity was larger than BW and ranged from 32.0% (B genome) to 88.2% (D genome) higher than that found in BW cultivars. The overall three-genome and the unanchored scaffold (ABD + unmapped) gene diversity of SHW (0.33) was 50.0% larger than that found in the BW cultivars (0.22).

**Table 5:** Population genetic diversity summary statistics of 101 synthetic hexaploid wheats (SHWs) and 12 bread wheat cultivars obtained from GenoDive

Genome	Population	No. of. Genotypes	SNPs used	Eff_num^a^	H_o_^b^	H_s_^c^	Gene diversity of SHW increased compared to BW (%)
A			11,297				
	Synthetic Hexaploid wheat	101		1.54	0.19	0.33	37.5
	Bread Wheat	12		1.38	0.17	0.24	
B			11,297				
	Synthetic Hexaploid wheat	101		1.54	0.19	0.33	32.0
	Bread Wheat	12		1.38	0.18	0.25	
D			10,008				
	Synthetic Hexaploid wheat	101		1.51	0.20	0.32	88.2
	Bread Wheat	12		1.26	0.17	0.17	
AB			23,930				
	Synthetic Hexaploid wheat	101		1.55	0.19	0.33	32.0
	Bread Wheat	12		1.38	0.17	0.25	
ABD			33,938				
	Synthetic Hexaploid wheat	101		1.54	0.19	0.33	50.0
	Bread Wheat	12		1.35	0.17	0.22	
ABD + unmapped			34,887				
	Synthetic Hexaploid wheat	101		1.54	0.19	0.33	50.0
	Bread Wheat	12		1.35	0.17	0.22	

^a^Eff-num: effective number of alleles, ^b^H_O_: observed heterozygosity, and ^c^H_S:_ gene diversity

## Discussion

### Population structure

The potential use of SHWs in genetic improvement of wheat for biotic and abiotic stresses resistance has been given a priority in many wheat breeding programs [7, 8, 10, 11, 35]. This study was designed to provide useful information regarding genetic diversity and population structure of SHWs that could potentially broaden the genetic base of BW germplasm as well help in GWAS to unravel unknown genomic regions or genes associated with economically important multiple traits.

In the present study, ~ 36,000 GBS derived high quality SNPs obtained from 101 SHWs were used. This study also demonstrates the usefulness of GBS derived SNPs markers for assessing the genetic diversity and population structure. The number of SNPs located on the A, B, and D genome in this study was in agreement with previous studies, where the B genome had the highest number of SNPs, followed by the A and D genome [13, 17]. Interestingly, in the present study, the number of SNPs on the D genome was similar to the number of SNPs on the A and B genomes. Generally, in previous studies, the number of SNPs in A or B genome is two [13, 14] to five [15, 16] times higher than in the D genome. This indicates that the SHWs have higher D genome sequence diversity than other sources. Greater sequence diversity (SNPs) of the D genome in SHWs may support the concept that the D genome has novel genetic variations and desirable genes [8] that can be utilized in elite wheat breeding program for broadening the genetic base. Broadening the genetic base may increase the rate of genetic gain, reduce the D-genome bottleneck, and help protect wheat from adverse effects of climate change due to currently limited genetic variation for key traits.

Two subgroups obtained from Bayesian clustering algorithm, neighbor joining cluster analysis, and PCoA were mainly divided based on the geographical location of the tetraploid (durum) parents (Romanian and Ukrainian durum in Winter_SHW group and USA durum in Spring_SHW group) rather than *Ae*. (diploid) parents. This result agreed with the results of Lage et al. [5], where SHW grouped together based on the geographical origin and presumed similar pedigrees of tetraploid parents. In SHW, two-thirds of the SHWs genome comes from tetraploid wheat (AABB) and one-third of the SHW genome comes from diploid parent (DD). Also, there were fewer durum parents (less diversity compared to *Ae. tauschii*) used in the SHW production which is the likely reason that SHW grouped together based on geographical location of tetraploid parent and growth habit of the crop.

Further population structure analysis was performed using Bayesian clustering algorithm for durum and *Ae.* parents separately to understand how they clustered. Durum parents were divided into two subgroups mainly based on the type/pedigree of durum parents used and its origin. When comparing two subgroups of durum parents to two subgroups of SHWs, all entries of Spring_Durum were in Spring_SHW and all entries of Winter_Durum were in Winter_SHW. Similarly, the *Ae.* parents also clustered into two subgroups. When comparing two subgroups of Aegilops parents to two subgroups of SHWs, all entries of Aegilops1 were in Spring_SHW and all entries of Aegilops2 were in Winter_SHW. Most of the entries of Aegilops1 were *Ae. tauschii ssp. strangulata* and most of the entries of Aegilops2 were *Ae. tauschii ssp. tauschii*. However, there was no distinct clustering based on the area of origin and type of the *Ae. taushii ssp*. Similar results were obtained in the past [28, 30]. For instance, in the study of a diversity panel of 322 *Ae. taushii*, *Ae. tauschii* were divided into four subgroups and the same *Ae. tauschii* ssp. or from the same area of origin were not clustered together (i.e., *Ae. tauschii ssp. tauschii* and *ssp. strangulata* did not separate entirely into separate clusters) [36]. This result may be potentially attributed to the event of migration leading to a decrease in genetic differentiation among subspecies [37] or wrong pedigree/classification information at the time of *Ae. tauschii* collections. However, in a different study of 477 *Ae. tauschii* accessions, *Ae. tauschii* were divided into two lineages (*Ae. tauschii ssp. tauschii* and *ssp. strangulata*) having little genetic overlap in the clusters [37].

### Genetic diversity

Analysis of molecular variation suggested that the population differentiation exists in two subgroups obtained from Bayesian clustering algorithm, where most of the variation was accounted by within population variance. Gene diversity (H_S_) for each subgroup showed that genetic variation in SHWs ranged from 0.18 (Spring_SHW) to 0.31 (Winter_SHW) with an overall gene diversity of 101 SHWs was 0.33. In Spring_SHW group, only one durum parent was used with different accessions of *Ae. tauschii* parents indicating that genetic variation observed within Spring_SHW was largely due to *Ae*. *tauschii* parents (D-genome diversity). Furthermore, the D genome gene diversity within 101 SHWs was 0.31 and genetic diversity of diploid parents (*Ae. tauschii*) from SHWs would expected to be very diverse and novel. The SHWs had a significantly higher level of gene diversity (H_S_ = 0.32 to 0.33) compared to elite BW cultivars (H_S_ = 0.17 to 0.25) in the present study. Similarly, higher gene diversity in SHWs have been reported in the past using AFLP [5] and SSR markers [6, 10, 38], indicating the usefulness of SHWs in introducing novel sources of genetic diversity into elite BW germplasm. For instance, gene diversity in 54 SHWs using AFLP marker was 0.39 [5]. Mean genetic diversity in SHWs using SSR markers reported in past were 0.5 [6], 0.61 [38], and 0.69 [10]. In general, the gene diversity of SNP makers is low due to its bi-allelic nature whereas SSR markers are high due to its multi-allelic nature. The gene diversity of SHWs using SNP makers in the past were lower than the present study. For instance, lower genetic diversity in SHWs compared to the present study was reported by Zegeye et al. [9], who evaluated 181 SHWs using 2590 SNP markers and found the genetic diversity ranged from 0.24 (B genome) to 0.26 (D genome).

The gene diversity of BW cultivars (10 cultivars from Nebraska, USA and two cultivars from Turkey) in our study ranged from 0.17 to 0.25. Similar results were obtained in a study of a diversity panel of 369 Iranian hexaploid wheat accessions. The gene diversity using SNP markers in Iranian wheat landraces and cultivars ranged from 0.14 to 0.20 [13]. The genetic diversity using GBS derived 20,526 SNPs in 8416 Mexican wheat landraces ranged from 0.06 to 0.26 [39]. The set of SHWs in our study had greater genetic diversity and was reported to have a multiple resistance to biotic and abiotic stresses [11]. This result suggests that the SHWs under study may provide a novel source of genetic diversity (novel alleles for a trait of interest) to the elite wheat breeding program.

## Conclusions

The present study provided a detailed understanding of genetic diversity and population structure of 101 SHWs and revealed high genetic diversity in the SHW compared to elite BW cultivars. Population structure analysis revealed that SHWs developed from diverse accessions of durum wheat and *Ae. tauschii* originated from different countries were divided into two (Spring_SHW and Winter_SHW) distinct groups based mainly on geographical location of durum parents and growth habit of the crop. Further population structure analysis of durum and *Ae.* parents separately identified two subgroups, mainly based on type/pedigree or origin of parents used. Although *Ae. tauschii* parents were divided into two groups mainly based on type of parent used, *Ae. tauschii ssp. tauschii* and *ssp. strangulata* did not separate entirely in each subgroup. The GBS derived SNPs were able to identify the inaccurate pedigree of synthetic hexaploid wheats based on misclassifications of some of the durum or *Ae.* parents from analyzing 139 SHWs and such misclassifications may have resulted due to heterozygous or heterogeneous parent lines, partial sterility and outcrossing, or seed handling error/mechanical mixing.

This study found that the percentage of SNPs on the D genome was nearly equal to that of other two genomes (A and B), which is unique to the SHWs as compared to previous studies on BW that reported that the D genome had less than 50% of the SNPs compared to A and B genomes. This result indicated the presence of high variation in the D genome in the SHWs. Furthermore, the gene diversity of SHWs under study was higher in all three genomes compared to elite BW cultivars and the greatest increase in gene diversity (88.2%) was observed in the D genome of SHWs compared to BW cultivars. Such higher genetic diversity in SHWs suggests that the diversity could be utilized in the elite wheat-breeding program to broaden the genetic diversity and increase genetic gain. The markers with high genome coverage such as GBS derived SNP markers are helpful for elucidating the population structure and genetic diversity. The results of this study will provide valuable information for wheat genetic improvement through inclusion of novel genetic variation and facilitate the discovery of novel source of genes/genomic regions conferring resistance to multiple biotic and abiotic stresses from association mapping study to unravel economically important marker-trait associations.

## Additional files

Additional file 1: Details of 139 synthetic hexaploid wheats used and subgroups obtained from Bayesian clustering algorithm for SHWs, durum wheat, and *Aegilops tauschii*. (XLSX 22 kb)

Additional file 2: Population structure analysis of 139 synthetic hexaploid wheats, durum and *Aegilops tauschii* parents obtained from Bayesian clustering algorithm. (DOCX 747 kb)

Additional file 3: Details of 101 synthetic hexaploid wheats (SHWs) used and subgroups obtained from Bayesian clustering algorithm for SHWs, durum wheat, and *Aegilops tauschii*. (XLSX 17 kb)

Additional file 4: Crossing scheme of 86-winter synthetic hexaploid wheat under study. (XLSX 10 kb)

Additional file 5: Kinship relationship matrix of 101 Durum wheat. (XLSX 116 kb)

Additional file 6: Kinship relationship matrix of 101 *Aegilops tauschii*. (XLSX 117 kb)

Additional file 7: Details of 12 bread wheat cultivars used in this study. (XLSX 9 kb)

Additional file 8: Genotyping-by-Sequencing derived SNP markers of 101 synthetic hexaploid wheats used in this study. (XLSX 15100 kb)

## Abbreviations

AFLP: Amplified Fragment Length Polymorphism
BW: Bread Wheat
CIMMYT: International Maize and Wheat Improvement Center
GBS: Genotyping-By-Sequencing
GWAS: Genome Wide Association Study
PCoA: Principal Coordinate Analysis
SHW: Synthetic Hexaploid Wheat
SNP: Single Nucleotide Polymorphism
SSR: Short Sequence Repeats (microsatellites)
