# Supplementary material for: Unlocking the novel genetic diversity and population structure of synthetic Hexaploid wheat
Source: BMC Genomics. 2018 Aug 6;19:591. doi: 10.1186/s12864-018-4969-2 (PMC6090860; doi:10.1186/s12864-018-4969-2)
Supplement: Supplementary file 2 — Population structure analysis of 139 synthetic hexaploid wheats, durum and Aegilops tauschii parents obtained from Bayesian clustering algorithm. (DOCX 747 kb) [file 12864_2018_4969_MOESM2_ESM.docx]

**Unlocking the Novel Genetic Diversity and Population Structure of Synthetic Hexaploid Wheat**

Madhav Bhatta, Alexey Morgounov, Vikas Belamkar, Jesse Poland, P. Stephen Baenziger*

Madhav Bhatta^1^, Alexey Morgounov^2^, Vikas Belamkar^1^, Jesse Poland^3^, P. Stephen Baenziger^1^*

^1^Department of Agronomy and Horticulture, University of Nebraska-Lincoln, Lincoln, NE 68583, USA

^2^International Maize and Wheat Improvement Center (CIMMYT), P.K. 39 Emek 06511, Ankara, Turkey

^3^Wheat Genetics Resource Center, Department of Plant Pathology, Kansas State University, Manhattan, KS, 66506, USA

*Corresponding author email: [pbaenziger1@unl.edu](mailto:pbaenziger1@unl.edu)

**Results regarding the population structure analysis of 139 synthetic hexaploid wheat**

One hundred thirty nine SHWs were analyzed for genetic diversity and population structure using 37,423 GBS derived SNPs (Additional file 1). Population structure analysis of 139 SHWs using ABD genome separated SHWs into three subgroups, mainly differentiated by geographical location of durum parents (Romania, Ukraine and USA) (Supplimentary figure 1.). Further population structure analysis of durum and *Ae.* parents separately identified four and three subgroups, respectively (Supplimentary figures 2 and 3). However, when looking at the grouping obtained for durum and *Ae.* parents, we found that some of the same durum parents or same *Ae*. parents did not cluster to their respective group based upon their given pedigrees. Therefore, in the new analyses, we removed 38 entries (five based upon the durum grouping and 33 based upon *Ae*. grouping) (Additional files 1 and 3). Their removal was based on misclassifications of either durum or *Ae.* parent lines into unexpected subgroups and segregation in the lines which was most likely due to heterozygous parent lines, or sterility and outcrossing within the SHW line (Additional file 1). For example, UKR 1530.94 durum was grouped together 48 times as expected but was not grouped with itself on one occasion leading us to think there was a mistaken pedigree, incorrect packaging of the seed, or something else occurred so that the line and its parentage are not what it was believed to be.  After removing 38 lines, 101 entries of SHWs were used for the genetic diversity and population structure analysis (Additional file 3).


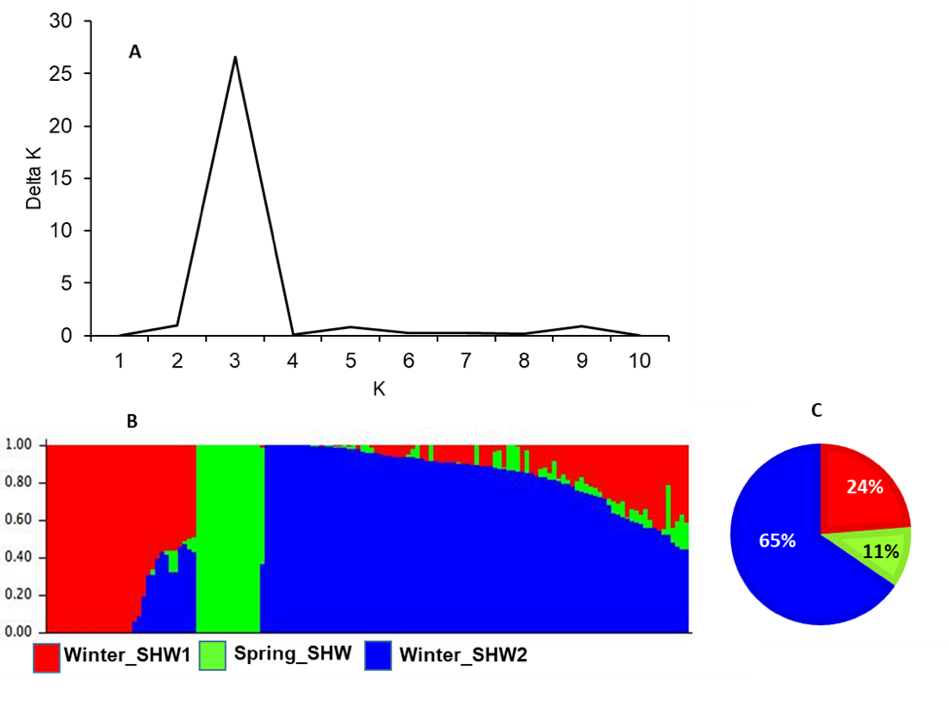


Figure S1 Population structure of 139 synthetic hexaploid wheats using the ABD genome. A: Line graph of delta K over K from 1 to 10, and the highest peak was observed at delta K=3, suggesting the synthetic hexaploid wheat (SHW) germplasm used in this study has three subgroups. B: Three subgroups were identified from the STRUCTURE and grouped based on the geographical location of the durum parents. C: Percentage of SHWs fell into each group i.e., 23% of SHWs in Winter_SHW1, 11% in Spring_SHW, and 66% in Winter_SHW2.

Figure S2. Population structure 139 synthetic hexaploid wheats using the AB genome (Durum parents). A: Line graph of deltak K over K from 1 to 10, and the highest peak was observed delta K=4, suggesting durum wheat has three subgroups. B: Four subgroups (Spring_Durum, Winter_Durum1, Winter_Durum2, and Winter_Durum3) were identified from the STRUCTURE.

Figure S3. Population structure of 139 synthetic hexaploid wheats using the D genome (Aegilops parents). A: Line graph of delta K over K from 1 to 10, and the highest peak was observed at delta K=3, suggesting the *Aegilops tauschii* (Aegilops) has three subgroups. B: Three subgroups (Aegilops1, Aegilops2, and Aegilops3) were identified from the STRUCTURE.
